# Otoprotection in guinea pigs exposed to pesticides and ginkgo biloba

**DOI:** 10.1590/S1808-86942012000400029

**Published:** 2015-10-20

## VOLUME 78 ISSUE 3 - MAY/JUN 2012

***Otoprotection in guinea pigs exposed to pesticides and ginkgo biloba***

*Andréa Dulor Finkler • Aron Ferreira da Silveira • Gisiane Munaro • Crisley Dossin Zanrosso*

**Figure 5:**
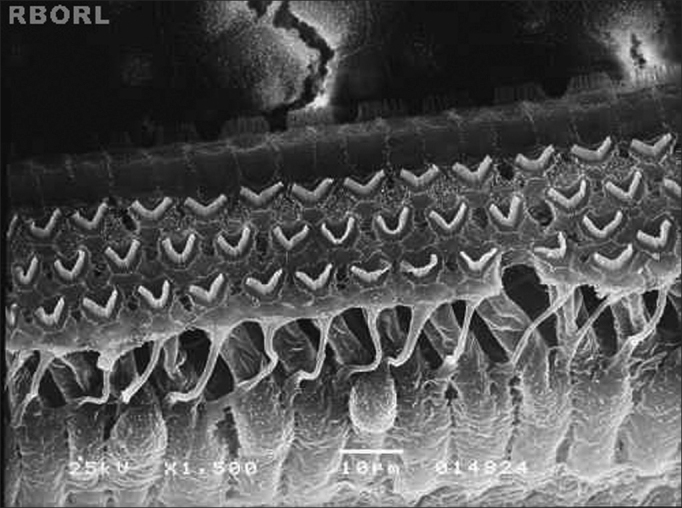
Photomicrography of the Corti's organ in a guinea pig of group 5, showing its basal turn. Observe the outer hair cells uninjured. Magnified 1500x.

Figure 5 was not printed on the following paragraph of page 126 of the English version of our Journal because of a technical issue:

“In group 5 (100mg/kg/day of Ginkgo biloba and, after 90 minutes, methamidophos 3,0 mg/Kg/day during 7 days), all the outer hair cells kept its normal shape and arrangement at the end of the experiment, as displayed in Figure 5.”

Below, the Figure is printed with its legend:

In the same paper, we include the following authors:

**Miguel Angelo Hyppolito** - PhD. Professor - Department of Ophthalmology, Otorhinolaryngology and Head and Neck Surgery - Medical School - Ribeirão Preto/USP.

**Adriana de Andrade Batista Murashima** - Biologist and Lab Technician - Medical School - Ribeirão Preto/USP.

